# Factors influencing mammography participation in Canada: an integrative review of the literature

**DOI:** 10.3747/co.v16i5.359

**Published:** 2009-09

**Authors:** K. Hanson, P. Montgomery, D. Bakker, M. Conlon

**Affiliations:** * School of Nursing, Laurentian University, Sudbury, ON; † Epidemiology, Outcomes and Evaluation Research, Regional Cancer Program, Sudbury Regional Hospital, Sudbury, ON

**Keywords:** Mammography, breast screening, Canada, integrative, review, barriers, facilitators

## Abstract

This integrative review critically examines quantitative and qualitative evidence concerning factors influencing the participation of Canadian women in mammography. Empirical studies published between 1980 and 2006 were identified and retrieved by searching electronic databases and references listed in published studies. Among the 1461 citations identified and screened, 52 studies met the inclusion criteria and were independently appraised by two researchers. Extracted data were categorized, summarized, compared, and interpreted within and across studies. The presentation of barriers and facilitators to mammography was guided by the Pender Health Promotion Model. Findings from this review showed that no published studies were specific to settings in Saskatchewan, Nova Scotia, Prince Edward Island, Newfoundland and Labrador, and the three Canadian territories. The most common barriers to screening were membership in an ethnic minority and concerns about pain, radiation, and embarrassment. The recommendation of a health care provider for mammography was found to be the most common facilitator for the engagement of women in this health behaviour. The targeting of specific strategies aimed at overcoming identified barriers and the enhancement of facilitators are essential to improving mammography participation rates throughout Canada.

## 1. INTRODUCTION

Breast cancer is the most common malignancy in women of all ages, accounting for 27.8% of all new cases and 15.1% of all cancer deaths in women 1. Mammography, a breast imaging technique, is the most common secondary prevention method. It has the ability to detect breast cancers at an early stage so that treatment is potentially more effective 2. A mammogram may be performed for a number of reasons, including screening for asymptomatic women, diagnostic assessment, follow-up for symptomatic women, and monitoring in high-risk groups.

Currently, organized breast screening programs are available in each Canadian province and two of the three territories 3. The population eligible for mammography screening is provincially determined and varies across Canada. For example, women in Ontario are eligible for screening at the age of 50; women in Alberta may enter the province’s organized screening program at 40 years of age. It is estimated that regular mammography screenings have the potential to reduce mortality rates by as much as 34% 4. According to Wadden and Doyle 3, federally established performance measures for Canada indicate that organized breast screening programs should screen at least 70% of the eligible population. This target was based on randomized controlled studies and is thought to be crucial for achieving a significant reduction in mortality rates 5.

Mammography services in Canada are available through organized breast screening programs, hospital diagnostic services, and private clinics. Approximately 60% of women between the ages of 50 and 69 have reported undergoing at least 1 mammogram from any of these three sources. Of these women, 34% participate in mammography through organized screening programs 3. Compared with other sources, organized screening programs are characterized by strong active recruitment efforts that often target hard-to-reach groups, by well-established reminder systems, and by the option for self-referral. These characteristics are all aimed at achieving the federally established target of 70% 2.

A vast amount of international and national literature pertaining to the challenges of recruiting women to breast screening is available. George 6 conducted an integrative review of 17 U.S. studies and identified various barriers for American women. The barrier to mammography screening most commonly reported was lack of physician referral. Other barriers included not believing that mammography was necessary, lack of knowledge, lack of time, inconvenience, procrastination, lack of available health care services, poverty, lower education, and ethnicity 6. From an Australian perspective, studies reported obstacles to screening such as ethnicity 7, mental illness 8, negative perceptions of mammography, inconvenience, lack of time, lack of awareness, and living in a rural setting 9. In two studies involving Israeli women 10,11, ethnicity, language difficulties, older age, not visiting a gynecologist, feelings of discomfort or embarrassment, a fatalistic attitude toward breast cancer, a mistrust of medical treatments, male practitioners, and travel were found to impede breast screening practices.

It is important to note that the reviewed literature did not always specify the type of mammography services studied or the purpose of the mammography procedure. Therefore, barriers and facilitators specific to the recruitment of women for screening mammography were not easily extracted from the published literature. Further, although the literature identified diverse factors negatively influencing mammography participation by women, it appears that such factors are not consistent across cultural groups. Published findings cannot be necessarily generalized to Canadian women, in part because of varying provincial health care practices or services and policies.

The present paper critically examines the empirical evidence about barriers and facilitators to mammography participation by Canadian women. The specific aims of this integrative review were to determine the extent and quality of the current literature base; to identify and describe barriers and facilitators influencing choice to participate in mammography; and to discuss clinical and research implications. Gaining a comprehensive understanding of the factors that influence the choice to participate in mammography can inform future recruitment efforts by organized screening programs throughout Canada. Through improved screening participation, reduced breast cancer mortality is possible.

## 2. MATERIALS AND METHODS

The design of this integrative literature review 12 was guided by a nursing systematic process of synthesizing and interpreting experimental and non-experimental published research. The findings from the included studies are combined into a narrative overview rather than converted into numeric data; the purpose is to enable answers to questions posed across numerous studies. The six phases of the review 12 are these:

- Problem identification
- Structured literature search
- Quality evaluation
- Data extraction
- Data analysis
- Presentation

The problem statement for the present review was “What are the identified barriers and facilitators to mammography participation for women living in Canada?”

**FIGURE 1:**
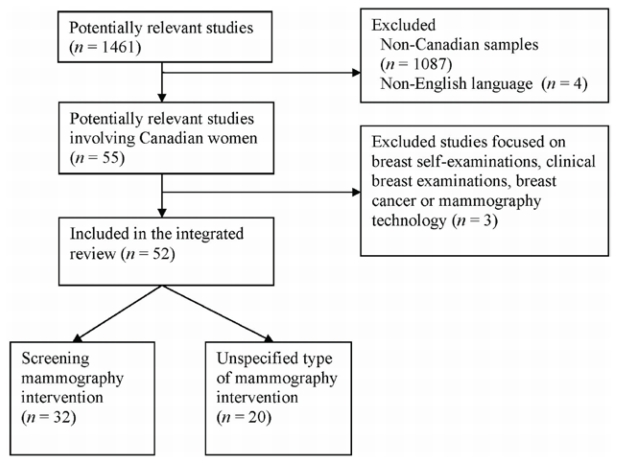
Flow diagram of the search-and-selection process for studies.

The literature search involved two strategies. First, we conducted a computerized literature search of electronic databases (medline, the Evidence-Based Medicine Reviews collection, PubMed, and cinahl) for January 1980 to December 2006. The search terms “mammography” and “breast screening” were independently combined with the key terms “barriers,” “recruitment,” “influencing factors,” “obstacles,” “challenges,” “facilitators,” “intervention,” and “strategy.” Second, we identified citations from reference lists in the studies located. Figure 1 shows the retrieval process.

The individual citations identified and reviewed for retrieval numbered 1461. Study selection was based on the following inclusion criteria:

- English-language publication
- Samples of Canadian women, regardless of age
- Investigation of variables impeding or enhancing (or both) initiation or adherence to mammography participation

Most citations were excluded for not meeting the foregoing criteria. If the relevance of a citation could not be ascertained from the abstract, the full article was reviewed. A total of 52 studies 13–64 were determined to be relevant for inclusion in the review.

Each of the 52 studies was independently appraised by two researchers using critical appraisal forms from the Joanne Briggs Institute 65. The Institute is an Australian evidence-based resource for nursing. Its 10-item standardized evaluation for health research requires the appraiser to assess the presence or absence of theoretic, methodologic, and methods information in each study. A reviewer responds to each item by scoring it no (“0”), yes (“1”), or unsure. A total study quality score is tabulated and can range from 0 (“low quality”) to 10 (“high quality”). Depending on the total appraisal score, the included articles were classified as low (1–3), medium (4–6), or high (7–10) quality.

After an appraisal of each quantitative study, data particular to factors influencing initiation or adherence to mammography were extracted if they showed statistically significant (*p* < 0.05) relationships to mammography participation. In qualitative studies, interpretations of data specific to mammography screening barriers and facilitators were identified. Extracted quantitative and qualitative findings alike were categorized, summarized, compared, and interpreted within and across studies. The Pender Health Promotion Model 66 was used to organize the various types of factors identified. From this perspective, individual, cognitive–perceptual, interpersonal, and situational variables contribute to an individual’s decision to participate in health-promoting behaviours —in this case, mammography screening. If an individual’s level of commitment to action outweighs competing demands or preferences, participation in a health-promoting activity is more likely. In contrast, the quantity and quality of competing demands may impede participation despite the individual’s intent to engage in the health behaviour 66.

## 3. RESULTS

### 3.1 The Quality and Extent of the Current Literature Base

Overall, most of the 52 studies received a high (*n* = 37, 71%) appraisal quality rating; the remaining studies were rated medium quality. Lack of a theoretic orientation, non-randomization of the sample, lack of generalization, and unknown reliability and validity of the data collection tools were common deficits in the quantitative articles. Despite 100% rater agreement on the quality category (high, medium, or low) of the included studies, rater agreement was lower (87%) with regard to the total quality scores of individual studies. The presence or absence of an articulated theoretic perspective was a frequent source of discrepancy between the two raters’ assigned total quality score for a study.

Table I presents the features of the included studies. Nearly 50% were published between 2001 and 2006, and most used a quantitative design. Most of the studies (62%) defined mammography as screening mammography; the remaining studies included screening and diagnostic mammography. In some studies, the reason for mammography intervention was not specifically stated. This omission may in part be a result of the evolution of population-based screening mammography programs in the late 1980s. Although organized breast screening has become more widely available throughout Canada, information about the type of mammography service or program was not specifically stated in many of the reviewed studies.

Mammography was most commonly implemented with Ontario women. Published research from Saskatchewan, Nova Scotia, Prince Edward Island, Newfoundland and Labrador, and the three Canadian territories was lacking. The age of the study participants varied greatly between the studies, ranging from under 40 years to more than 80 years of age. Sixteen investigations (31%) included women not actively targeted by provincial programs. The sample sizes ranged between 15 and 572,762 women. In nine studies (17%), samples of minority women were included. Approximately one third of the studies undertook a quantitative secondary analysis using national or provincial health surveys.

### 3.2 Barriers to Mammography Screening

Table II presents the barriers to mammography screening, in accordance with the Pender categories of health promotion factors 66.

At an individual level, barriers for Canadian women included a woman’s past and present behaviours, her personal attributes, and her socioeconomic status. With regard to behaviour, Canadian women were less likely to attend breast screening if they did not participate in other screening behaviours such as clinical breast examinations, blood pressure checks, or Pap smears. They were also less likely to be screened if they smoked or infrequently participated in healthy lifestyle behaviours such as exercise. Personal attributes associated with lack of screening included membership in an ethnic minority, older age (the particular ages identified varied depending on the sample used in each study), being less informed or knowledgeable about breast screening or breast cancer, having language or communication difficulties, or living in a rural area. Women with a previous abnormal mammography result were also less likely to be screened through a screening mammography centre because of a suggested need for diagnostic mammography. Finally, women in low income brackets according to Canadian census parameters were also identified as having lower rates of mammography participation. Discrepancies existed with regard to educational attainment. Four studies (8%) identified lower educational attainment (high school or less) and one study (2%) identified higher educational attainment (postsecondary) as barriers for Canadian women. Researchers suggested that this discrepancy may be a result of enhanced participation of the lower-education group after implementation of target-specific recruitment interventions.

Cognitive–perceptual barriers are factors associated with the individual’s thoughts and feelings. For Canadian women, cognitive barriers included negative perceptions of mammography or breast cancer and negative or positive perceptions of self. Negative perceptions of mammography have been identified as concerns about the procedure, radiation exposure, or pain; not believing that mammography is necessary or effective; believing that mammography isn’t a priority; and feelings of embarrassment or modesty. Negative perceptions of breast cancer include a fear of finding something wrong and being pessimistic regarding breast cancer survival. Believing that there is a stigma associated with cancer was also identified as a barrier in some ethnic minority groups. For these women, screening may be avoided to minimize the risk of knowing that they have cancer. Negative perceptions of self included low self-esteem, and positive perceptions of self included feeling healthy.

Interpersonal barriers are the influences of others on an individual’s decision to participate in mammography. For Canadian women, limited physician access, lack of a screening recommendation, or lack of health care provider support was identified in a number of studies. An intimidating physician–patient hierarchical relationship was also identified and may prevent some women from accessing information on breast health. Finally, limited social support was also associated with reduced participation.

Situational barriers are factors in the environment that inhibit mammography screening. Situational barriers identified in the Canadian literature included health service deficiencies and negative health messages. Health service deficiencies reflect a lack of physician reminder systems. Negative health messages included conflicting information about screening and fearful depictions of breast cancer in the media.

### 3.3 Facilitators to Mammography Screening

Table III presents the groups of facilitators to mammography screening participation in the Canadian literature.

Facilitators at the individual level included a woman’s past and present behaviours, her personal attributes, and her socioeconomic status (notably also identified as barriers). With regard to behaviours, women who participated in other screening behaviours (clinical breast examinations, Pap tests, previous mammograms, blood pressure checks), who engaged in healthy lifestyle behaviours such as refraining from smoking, and who used female hormones were more likely to have a mammogram. Participation in breast screening was also increased for women who were socially involved in activities such as volunteerism, associations, and church. Personal attributes associated with enhanced screening included age between 50 and 69 years, proficiency with the English language or bilingualism, birth outside of Canada, a higher body mass index, being married or having a partner, nulliparity or first birth at a later age, having a family history of breast cancer, being well informed or knowledgeable about breast cancer or screening, having a normal initial screen, and urban or rural residency. Finally, women with higher or adequate income, private insurance in addition to universal coverage, postsecondary education or higher, and current employment also had improved screening rates.

Cognitive facilitators have been categorized as negative perceptions and positive perceptions. Enhanced screening was associated with negative perceptions such as a perception of breast cancer risk and a fear of cancer. It was also associated with positive perceptions such as feelings of personal well-being, no prior breast pain, the absence of negative attitudes, and low decisional conflict.

Interpersonal-level facilitators involved support or encouragement from the health care community or significant others. Examples of encouragement from the health care community included physician recommendation, encouragement from nurses, recent physician visits or regular physician care, and having a female health care provider. A woman’s informal network of friends, family, and co-workers was also influential, especially when screening was an accepted health practice.

Finally, within the Canadian literature, situational facilitators included exposure to the screening message and adequate screening services. Exposure to the screening message included the use of media, literature, pamphlets, and videos. It also involved the use of multiple promotional sources to relay the health message. Adequate screening service included breast screening services that were sensitive to a woman’s health needs; combinations of courteous, competent, and prompt service that included teaching; the use of patient reminders; mass screening programs; and a tailored curriculum directed at physicians.

### 3.4 Competing Demands Identified in the Canadian Literature

According to Pender *et al.* 66, the barriers and facilitators present in a woman’s life influence her level of motivation to participate in screening. Competing demands or preferences have the potential to impede participation even when intent exists.

A few competing demands were identified in the present review of the literature (see Table II). They include lack of time or “not getting around to it,” perceptions that the service is inconvenient or difficult to access, and excessive distance or transportation difficulties. If a woman’s level of motivation or intent outweighs the existence of these competing demands, the likelihood of participation in mammography screening is potentially enhanced.

## 4. DISCUSSION

This review includes 52 Canadian studies. Most of our knowledge of factors influencing mammography participation has been derived from various quantitative studies using populations of women living in Ontario. The research available for populations of Canadian women living in Saskatchewan, Nova Scotia, Prince Edward Island, Newfoundland and Labrador, and the three Canadian territories was limited. Some of these populations were represented in studies that utilized the National Population Health Surveys; unfortunately, those surveys excluded from their samples women residing in the territories. Further research is recommended to determine if women living in these northern Canadian regions experience unique contextual or cultural challenges to mammography participation.

Research pertaining to the unique barriers and facilitators for various ethnic groups throughout Canada was also limited. Although many quantitative investigations have identified ethnicity and language barriers as factors impeding mammography participation, few in-depth qualitative investigations were found to further an understanding of those barriers. The qualitative studies included in this review have contributed to our understanding of unique ethnic perspectives, but future qualitative research is recommended. Enhanced knowledge of influencing factors for Canadian minority groups would inform recruitment efforts targeting other ethnic communities. Given that the most common barrier to breast screening identified in the Canadian literature was membership in an ethnic minority, individual organized programs must remain diligent in their efforts to target women from the specific ethnic groups in their regions. This targeting may be facilitated by the development of collaborative partnerships with knowledgeable community leaders. Such partnerships may provide breast screening programs throughout Canada with an avenue to increase awareness of cultural concerns and may enhance the provision of services in a way that is sensitive to cultural practices and beliefs 67. Individual-directed strategies such as culturally appropriate mailed letters and reminders, in-person or telephone counselling, and specifically targeted print material may be effective at overcoming the unique barriers associated with cultural beliefs and practices. Increases in mammography participation have been associated with individual-directed interventions that relay health information using a tailored approach 68.

Other common barriers for Canadian women included concerns of pain, radiation, and embarrassment. Negative attitudes regarding mammography were also identified as barriers for populations in other countries 6,9. Individual-directed strategies, media campaigns, and social networking interventions designed to ease such concerns may also be of benefit 68.

In an integrative review of the American literature, George 6 identified low income and lack of physician referral as factors impeding mammography participation in the United States. It is interesting to note that low income and lack of physician recommendation remains a barrier for Canadian women despite the availability of free mammograms and self-referral. In fact, in the present review, the most common facilitator to mammography screening for Canadian women remains a recommendation from a health care professional such as a doctor or nurse. Despite the availability of self-referral, organized programs must continue to educate the health care community concerning the importance of their support. Provider- and system-directed strategies such as computerized and manual prompting systems aimed at facilitating professional recommendation may also be of benefit 68.

A number of the barriers and facilitators to mammography screening participation identified in this review could inform recruitment efforts by screening programs. Because organized screening programs throughout Canada rely on limited publicly funded resources, administrators must direct their funding dollars toward efforts with the greatest potential for success. It is therefore recommended that strategies targeting specific populations of women or the health care community be evaluated to ensure that public dollars are being spent in the most efficient and effective manner. To facilitate enhanced recruitment, further research should also be conducted to explore whether the designed interventions were perceived as acceptable and appropriate by the target population. Results of such studies could inform future recruitment efforts.

Common limitations were identified in the articles reviewed here. The lack of randomization, which limits generalization beyond the study samples, was identified in many of the quantitative studies 69. Barriers and facilitators identified in the studies may not be representative of the eligible female population in Canada. Most of the current literature is based on research that used a descriptive or cross-sectional design. These studies provide insight into perceptions and practices, but they prevent any assumption of causality 69. Finally, a number of studies relied on older survey data. One survey explored physician perceptions in Quebec before 1983 17 and three studies 32,47,64 were based on data obtained from the 1990 Ontario Health Survey. It is important to note that these surveys were undertaken before the establishment of organized screening programs in Ontario and Quebec. A different health care climate and changing perceptions may have rendered these results outdated.

### 4.1 Limitations

Although efforts were made to conduct a thorough review of the literature, it is possible that some of the available literature may not have been retrieved. This potential exists because of inconsistencies in search terminology, indexing problems, and publication bias 12. Our review was restricted to articles written in the English language, which may explain why research from the province of Quebec was limited. The use of a structured tool for the assessment of study quality was a strength in our review, but we found the application of the quality assessment tools challenging. Certain study deficits resulted in a more pronounced loss of quality points. For example, studies that used a nonrandomized sample were penalized twice, once for the chosen sampling technique and again for results that were not generalizable. This case also held for articles that did not outline their guiding theoretic framework. A quality mark was lost for not identifying a theory, and an additional mark was lost if the findings were not linked to a guiding framework. Finally, because of publication restrictions, certain methodologic aspects covered by the tool may not have been included in the published article, resulting in a lower quality score.

## 5. CONCLUSIONS

Regular mammography screenings have the potential to reduce breast cancer mortality rates. The present integrative review identified a number of barriers to mammography participation for women living in Canada. Various facilitators to participation that may guide the recruitment efforts of organized breast screening programs were also identified. Because research regarding mammography screening for certain populations of women living in Canada is limited within the current literature base, future research is recommended.

## Figures and Tables

**TABLE I:** Summary of the Canadian literature

Study characteristics	Studies (n of 52)	References to the included studies
Study dates		
1980–1985	1	17
1986–1990	1	14
1991–1995	4	21,41,49,52
1996–2000	19	15,18,20,22–26,29,31,32,36,37,39,43,47,53,61,64
2001–2006	27	13,16,19,27,28,30,33–35,38,40,42,44–46,48,50,51,54–60,62,63
Study types		
Quantitative	46	13–19,21,22,24–26,28–45,47–53,55–57,59,60–64
Qualitative	4	20,23,27,58
Mixed methods	2	46,54
Mammography type		
Exclusively screening	32	13–15,17–22,24,26–28,33,34,36,40–42,46,48,49,52,54–61,63
Unknown or mixed types	20	16,23,25,29–32,35,37–39,43–45,47,50,51,53,62,64
Study location^a^		
Canada in general	17	16,23,25,29,35,37,40,43–45,50,51,53,58,59,61,62
British Columbia	5	20,27,33,36,60
Alberta	2	21,41
Saskatchewan	0	
Manitoba	2	14,42
Ontario	21	13–15,19,22,24,26,28,30–32,38,39,46,47,49,52,54,57,63,64
Quebec	4	17,18,34,55
New Brunswick	2	48,56
Nova Scotia	0	
Prince Edward Island	0	
Newfoundland and Labrador	0	
Canadian Territories	0	
Sample type^a^		
Canadian women		
40 years of age and older	32	14–16,18,19,21,22,24–27,29,30,33–38,41,43–46,48,49,52,54,56,57,60,62
including those <40 years of age	16	20,28,31,32,39,40,42,47,50,51,53,58,59,61,63,64
Health care professionals	5	13,17,27,46,55
Members of minority groups	9	20,22,27,33,46,51,54,56,60
Sample size		
<50	2	27,46
50–100	5	20,22,23,42,58
101–1000	18	13,15,17–19,24,26,33,39,48,49,52,54–56,59,60,63
1001–100,000	24	14,16,21,25,28,29,32,35–38,40,41,43–45,47,50,51,53,57,61,62,64
>100,000	3	30,31,34
Data collection method or source^a^		
Secondary analysis of population health surveys	18	16,25,28,29,32,35,37,38,43–45,47,50,51,53,61,62,64
Cross-sectional surveys	25	13–15,17–19,21,22,24,26,33,36,39,40,41,42,46,48,49,52,54,56,59,60,63
Databases (government, screening program)	7	24,28,30,31,34,38,57
In-depth interviews	4	20,27,54,58
Focus groups	1	46
Document analysis	1	23

a Multiple categories may have been used.

**TABLE II:** Barriers to screening identified in the Canadian literature

Barrier category	Barrier	Studies (%)	References
Individual level	Ethnic minority	19	20,27,29,30,43,44,46,47,51,54
	Older age	13	16,19,29,31,39,41,44
	Low income/SES	8	30,43,47,57
	Lower educational attainment	8	38,43,47,53
	Lack of knowledge	6	41,52,58
	Smoking	6	43,44,48
	Communication difficulty	6	20,46,47
	Rural residency	6	21,43,44
	Infrequent exercise	4	43,44
	Lack of previous screening behaviours	4	43,47
	Previous abnormal result	2	57
	Higher educational attainment	2	16
Cognitive–perceptual level	Concerns about radiation/harm	15	13–15,17,26,41,48,49
	Pain/discomfort	12	13–15,26,41,49
	Embarrassment or modesty	10	13,20,26,27,46
	It’s not necessary	10	13,26,41,44,49
	It’s not a priority	8	14,46,48,58
	It’s ineffective	6	13,17,49
	Fear of the procedure	4	18,52
	Fear of finding something wrong	4	26,49
	Pessimism regarding breast cancer	4	20,52
	Stigma	4	20,46
	Feeling healthy	2	48
	Low self esteem	2	43
Interpersonal level	Lack of physician recommendation	8	14,26,41,49
	No regular doctor or recent doctor’s visit	6	29,43,44
	Limited social support or encouragement	6	14,43,52
	Physician–patient hierarchy	2	27
Situational level	Conflicting information about screening	4	14,23
	Fearful depictions of breast cancer	4	14,23
	Lack of a physician reminder system	2	13
Competing demands	Lack of time	8	13,14,18,48
	Service is inconvenient	6	14,40,49
	Excessive distance/travel difficulties	6	14,40,49
	Not getting around to it	4	44,49
	Service difficult to access	2	17

SES = socioeconomic situation.

**TABLE III:** Facilitators to screening identified in the Canadian literature

Facilitator category	Facilitator	Studies (%)	References
Individual level	Older than 50, but younger than 70	15	19,29,35,43,44,53,57,62
	Participation n other screening behaviours	13	16,18,19,35,48,49,57
	Increased income/SES	13	28,32,37,49,50,60,61
	Increased educational attainment	12	16,28,29,33,37,42
	Being married or having a partner	12	24,29,33,43,50,60
	Healthy lifestyle behaviours	8	16,18,32,35
	Proficiency with English language	8	22,33,57,64
	Use of female hormones	6	16,43,47
	Being well informed about screening	6	33,49,63
	Social involvement	4	38,43
	Being employed	4	29,60
	Older age	2	61
	Being bilingual	2	43
	Being born outside of Canada	2	16
	Higher BMI	2	50
	Family history of breast cancer	2	56
	Urban residency	2	16
	Rural residency	2	57
	Nulliparous or first birth at a later age	2	36
	Normal initial screen	2	36
	Having private insurance	2	60
Cognitive–perceptual level	Perception of breast cancer risk	8	24,33,48,63
	Fear of cancer	2	52
	Feelings of well-being	2	47
	No prior breast pain	2	36
	Low decisional conflict	2	49
Interpersonal level	Doctor/nurse recommendation	27	18–21,26,33,36,39,40,52,56–59
	Recent or regular physician care	15	28,35,45,47,50,60–62
	Encouragement from friends, family, co-workers	15	19,20,26,33,39,40,52,59
	Having a female health provider	4	33,60
	Encouragement from well known individuals	4	39,59
Situational level	Media advertisement	8	33,39,58,59
	Literature/pamphlets/videos	8	33,39,58,59
	Courteous, competent service	6	14,40,58
	Patient reminders	4	34,58
	A program that is sensitive to a woman’s health needs	2	58
	Mass breast screening programs	2	25
	Tailored curriculum for doctors	2	55

SES = socioeconomic situation; BMI = body mass index.
